# Proton Transport in Aluminum-Substituted Mesoporous Silica Channel-Embedded High-Temperature Anhydrous Proton-Exchange Membrane Fuel Cells

**DOI:** 10.1038/s41598-020-66935-5

**Published:** 2020-06-25

**Authors:** Kwangwon Seo, Ki-Ho Nam, Haksoo Han

**Affiliations:** Department of Chemical and Biomolecular Engineering, Yonsei University, 50 Yonsei-ro, Seodaemun-gu, Seoul 120-749 Republic of Korea

**Keywords:** Chemical engineering, Materials for energy and catalysis, Fuel cells

## Abstract

Polymer composite membrane technology is promising for enhancing the performance of membrane electrode assemblies for high-temperature fuel cells. In this study, we developed a novel anhydrous proton-exchange polybenzimidazole (*m*-PBI) composite membrane using Al-substituted mesoporous silica (Al-MCM-41) as a proton-carrier support. The surface-substituted Al-MCM-41 formed effective proton-transport pathways via its periodic hexagonal channel and improved the proton conductivity. The proton conductivity of an *m*-PBI filled with 9 wt.% filler was 0.356 S cm^-1^ at 160 °C and 0% humidity, representing an increase of 342% compared to that of a pristine *m*-PBI. Further, the current density at 0.6 V and maximum power density of *m*-PBI composite membranes were increased to 0.393 A cm^-2^ and 0.516 W cm^-2^, respectively. The enhanced fuel-cell performance was attributed to the proton-transfer channels and H_3_PO_4_ reservoirs formed by the mesopores of the Al-MCM-41 shell. The results indicated that Al-MCM-41 is suitable with respect to the hybrid homologues for enhancing the proton transport of the *m*-PBI membrane.

## Introduction

Over the past few years, the direct conversion of chemicals into electrical energy through fuel cells has attracted considerable attention in electrochemical research and technology development^1–3^. This is not only owing to the scientifically fascinating complexity of fuel-cell reactions and the technological potential of fuel cells but also a result of society’s efforts to achieve ecofriendly power generation. The polymer electrolyte membrane fuel cell (PEMFC) was the first type of fuel cell to be practically applied—it provided onboard power for NASA’s Gemini spaceship in 1960^4^. The PEMFC is now regarded as a promising alternative power source for automotive transportation, portable power, and power generation applications^5–7^. The advantage of the PEMFC over other fuel cells (e.g., alkaline fuel cells, phosphoric acid fuel cells, molten carbonate fuel cells, and solid oxide fuel cells) is that it can generate power density at the lowest operating temperature.

In recent years, industrial and academic research on the PEMFC has focused on the optimization of devices operating at temperatures above 100 °C to increase the system efficiency^8–13^. High-temperature polymer electrolyte membranes (HT-PEMs) operating above 100 °C without humidification offer many benefits, including fast electrode kinetics, a high tolerance to fuel impurities such as carbon monoxide, and a simplified system design^14–16^. Hence, considerable effort has been directed towards the development of low-cost, high-performance, and high-temperature-resistant alternative hydrocarbon-based polymer electrolyte membranes (PEMs) for high-temperature-operating PEMFCs^17–20^. Acid-doped polybenzimidazole (PBI), which was introduced by Savinell *et al*.^15,21,22^, is fascinating and remains the most important polymer membrane for high-temperature polymer electrolyte membrane fuel cell (HT-PEMFC) applications because of its low fuel crossover and good electrochemical properties at high temperatures (up to 200 °C) and under anhydrous conditions^23–28^. However, PBI membranes have limitations (the most important ones are the acid leaching from the membranes and the mechanical instability during high-temperature operation); thus, they are currently not a viable replacement for Nafion-based membranes.

Polymer composite membrane technology is promising for enhancing the fuel-cell performance under high-temperature operating conditions. Substantial effort has been directed towards the development of acid-doped PBI membranes reinforced by nano- and mesoporous materials (e.g., silica^29–31^, titanium oxide^32–34^, zeolite^35^, and solid acid^19,36^) in recent years. The main challenge for composite membranes is the harmonious architecture design of the proton-conducting groups, proton-transport channels, and acidic reservoirs. Our group have previously demonstrated the positive effects of aluminum (Al)-substitution of spherical silica nanoparticles on the proton transfer^37^. In the present study, the introduction of an Al-substituted hexagonally ordered mesoporous silica (Al-MCM-41) channel into a poly(2,2′-m-(phenylene)-5,5′-bibenzimidazole) (*m*-PBI) membrane significantly enhanced the cell performance and durability at the high end of the operating-temperature regime. Stable operation was achieved under dry conditions at 150 °C for 600 h, which is a significant milestone in HT-PEMFC development.

## Results and discussion

**Figure 1:**
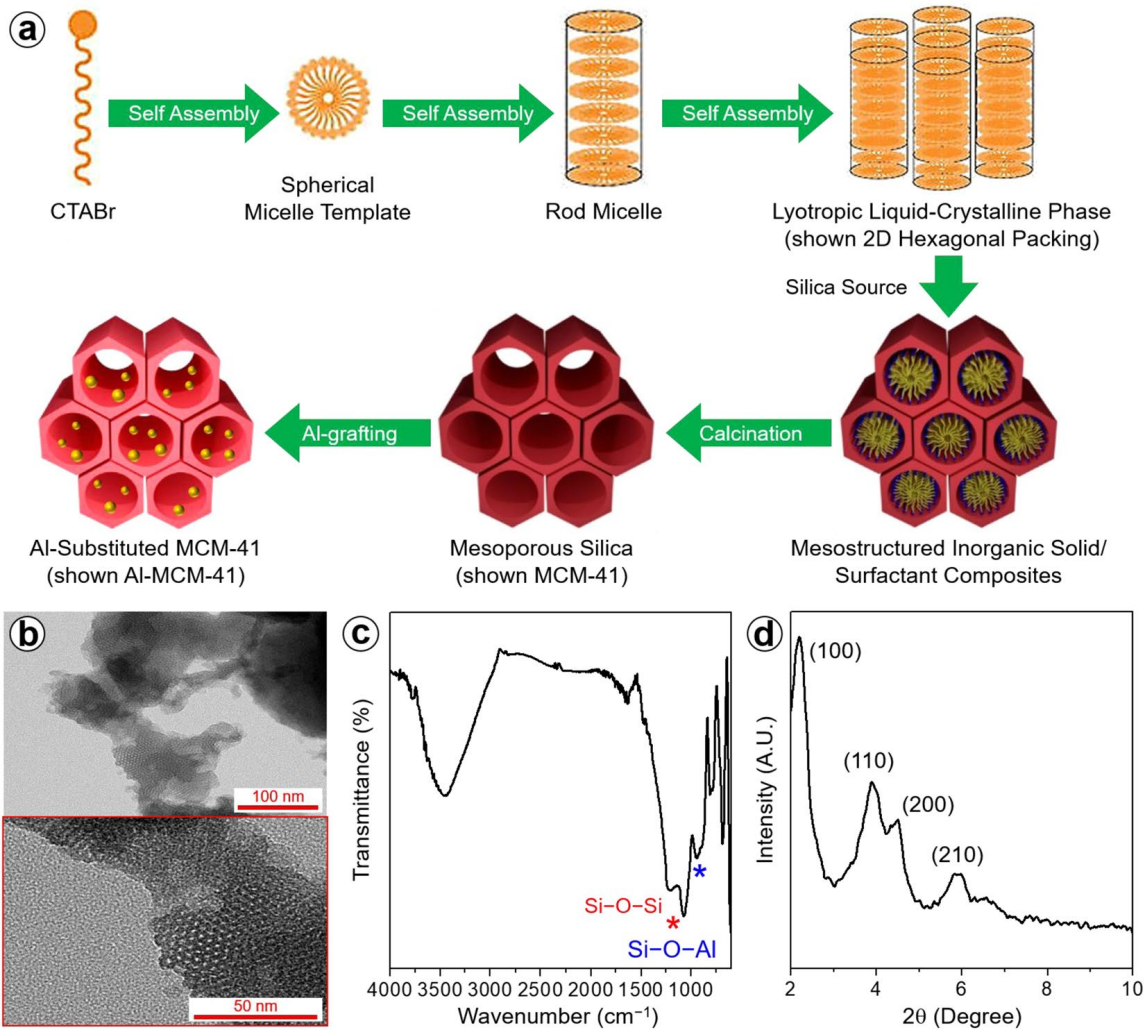
(**a**) Preparation scheme, (**b**) TEM image, (**c**) FTIR spectrum, and (**d**) XRD pattern of Al-MCM-41.

The main objective of this study was to develop new organic–inorganic composite membranes that are applicable to the HT-PEMFC. The incorporation of a proton-carrier support channel within the membrane is considered effective for increasing the H_3_PO_4_ uptake and proton conductivity and enhancing the high-temperature durability. Al substitution of mesoporous silica was achieved via the sol-gel reaction, followed by surface grafting of Al, as shown in Fig. 1(a). TEM images (Fig. 1(b)) show that the Al-MCM-41 had a well-ordered honeycomb patterned porous structure. The hexagonal straight channels extended along the one-dimensional particles. The chemically direct connection of the Al with the framework surface of the MCM-41 was investigated via FTIR spectroscopy, as shown in Fig. 1(c). We assigned the peaks at 1050 cm^−1^ to the Si–O − Si stretching vibration. The symmetrical and stretching vibration peaks of C − H appeared at 710 and 810 cm^−1^, respectively. Notably, the absorption peak for Si−O − Al at 800 cm^−1^ was observed as a result of the grafting of Al onto mesoporous silica, in accordance with a previous report of Al surface sites grafted onto silica^38,39^. The XRD pattern of Al grafted onto MCM-41 exhibited crystalline peaks corresponding to the (100), (110), (200), and (210) planes (in the narrow-angle profile) (Fig. 1(d))^40^.

**Figure 2:**
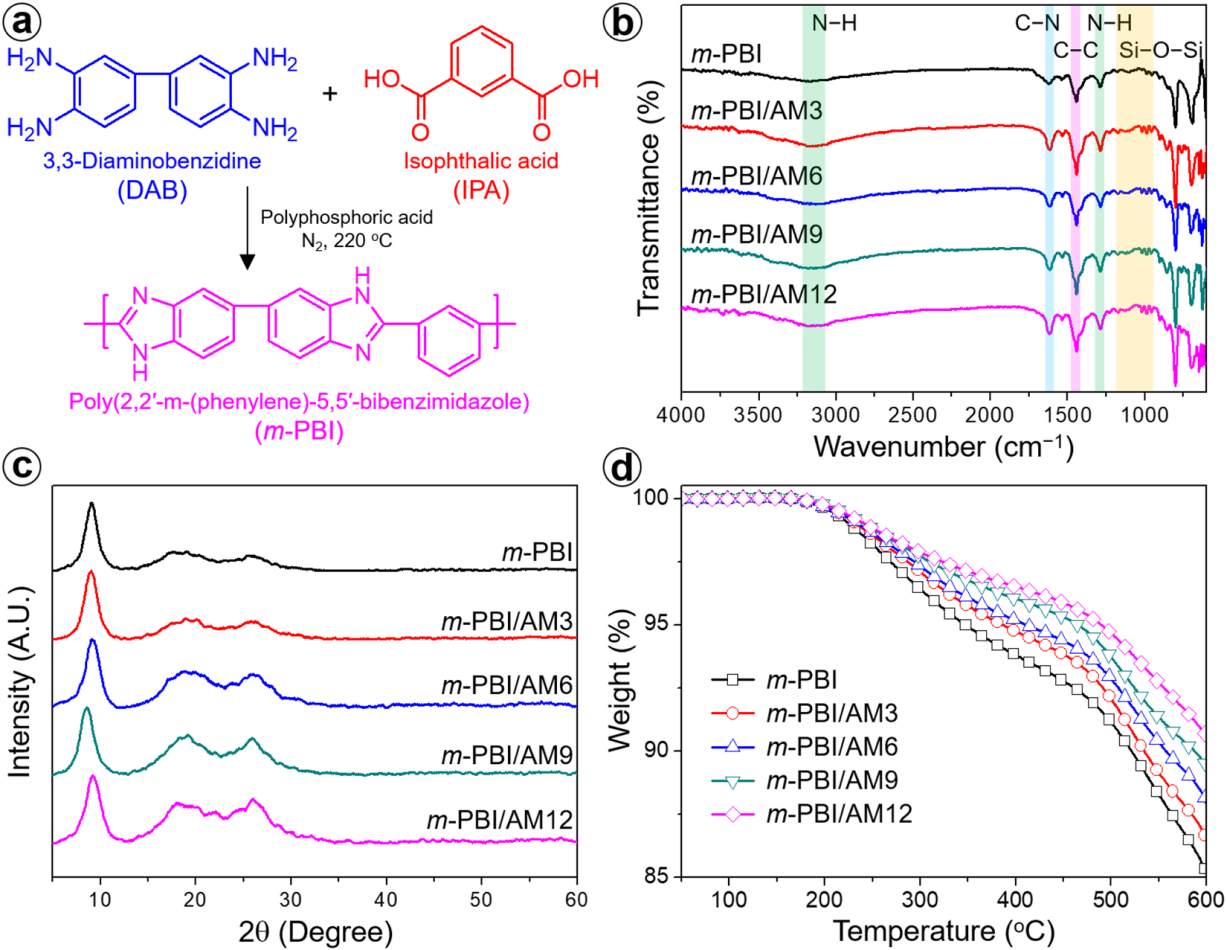
(**a**) Reaction for the synthesis of *m*-PBI, (**b**) FTIR spectra, (**c**) XRD patterns, and (**d**) TGA thermograms of *m*-PBI/Al-MCM-41 composite membranes.

Acid–polymer combination is an effective approach for the development of proton-conducting membranes. As a proton conductive binder, the synthetic scheme and the chemical structure of poly(2,2′-m-(phenylene)-5,5′-bibenzimidazole) (*m*-PBI) are shown in Fig. 2(a). Figure 2(b) presents the FTIR spectra of *m*-PBI composite membranes with different Al-MCM-41 loadings, which indicate their chemical compositions. The pristine *m*-PBI and the composites exhibited the characteristic absorption peaks of N − H and aromatic C − C at 1300 and 1450 cm^−1^, respectively. The benzimidazole group was confirmed by peaks corresponding to imine stretching at 1630 cm^−1^ and N − H hydrogen bonding at 3200 cm^−1^. The characteristic absorption peaks at 1190, 1050, and 800 cm^−1^ for the *m*-PBI/Al-MCM-41 composite membranes corresponded to the asymmetric and symmetric stretching of the Si−O − Si bonds, which is consistent with the results obtained by David Aili *et al*.^41^. Furthermore, a stretching vibration peak at 610 cm^−1^ corresponding to the Al−O bonds was clearly observed for the composite membranes. Figure 2(c) presents the XRD patterns of *m*-PBI composite membranes with different Al-MCM-41 compositions. The XRD patterns of all the membranes exhibited a distinct diffraction peak centered at 2θ = 10.1°, corresponding to a *d*-spacing (average inter-segmental distance between chains) of 8.8 Å, along with several small peaks at 2θ = 19.4 and 25.1°. The *m*-PBI/Al-MCM-41 composite membranes exhibited an additional peak at 2θ = 24.8° and no significantly shifted peaks, indicating an improved molecular order and structural regularity in the amorphous PBI polymer. To evaluate the H_3_PO_4_ doping level, the thermal-degradation behavior of the acid-doped *m*-PBI composite membranes was investigated, as shown in Fig. 2(d) and Table S1. As indicated by Fig. 2(d), the membranes started to degrade at approximately 200 °C, even though the boiling point of H_3_PO_4_ is 158 °C. This indicates that a favorable interaction between the Al-substituted mesoporous channel/imidazole group complex and H_3_PO_4_ occurred, preventing the degradation of H_3_PO_4_. With the increasing Al-MCM-41 loading amount, the 5% and 10% degradation temperatures decreased slightly because the amount of H_3_PO_4_ in the acid-doped membrane increased. A lower degradation temperature corresponds to more extensive degradation of H_3_PO_4_; thus, the doping amount of H_3_PO_4_ increased with the Al-MCM-41 content^42^. Furthermore, the amount of carbonized residues at 600 °C decreased with increasing filler loading amount. The correlation between the Al-MCM-41 concentration and the H_3_PO_4_ doping amount was confirmed via doping level testing, and the relationship between the doping amount and the membrane performance was investigated.

**Figure 3:**
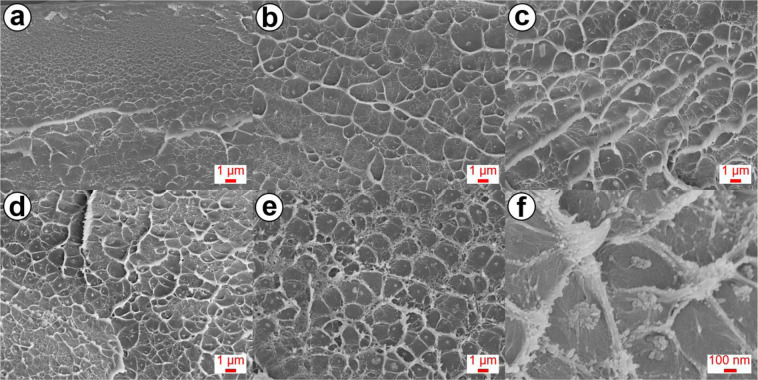
Cross-sectional SEM images of *m*-PBI/Al-MCM-41 composite membranes with different weight loadings of Al-MCM-41. (**a**) pristine, (**b**) 3 wt.%, (**c**) 6 wt.%, (**d**) 9 wt.%, and (**e**) 12 wt.%. (**f**) Magnified image of the *m*-PBI/AM12 membrane.

The dispersion state of Al-MCM-41 in the PBI matrix was investigated in detail using SEM cross-sectional images. The pristine *m*-PBI exhibited a continuous phase (Fig. 3(a)), whereas the composite membranes exhibited homogeneously distributed domains (Fig. 3(b–e)). The well-dispersed Al-MCM-41 channels allowed the *m*-PBI composite membrane to hold more H_3_PO_4_, increasing the H_3_PO_4_ doping level, which was directly related to the proton conductivity and MEA performance. However, several agglomerates of fillers were observed in the *m*-PBI/AM12 (Fig. 3(f)). This phenomenon can affect the formation of cracks and voids in the composite membranes, negatively affecting the properties (e.g., durability) of the membranes^43,44^.

When the acid-doped membranes were prepared via the direct method, their doping level could be higher than that of membranes prepared via the conventional process. The PA doping amount, doping level, and calculated values are presented in Table S2. The amount of PA per specific volume and acid doping level of the composite membranes increased compared to pristine *m*-PBI. Specifically, the amount of H_3_PO_4_ per specific volume and H_3_PO_4_ doping level of the *m*-PBI/AM12 membrane were approximately five and three times greater, respectively, than those of pristine *m*-PBI. This trend is attributed to the special structure of tunnel arrays of Al-MCM-41, which allowed more H_3_PO_4_ to be held inside the filler, and the strong attraction with H_3_PO_4_ owing to the Al atoms on the filler surface (see Supplementary Fig. S3).

**Figure 4:**
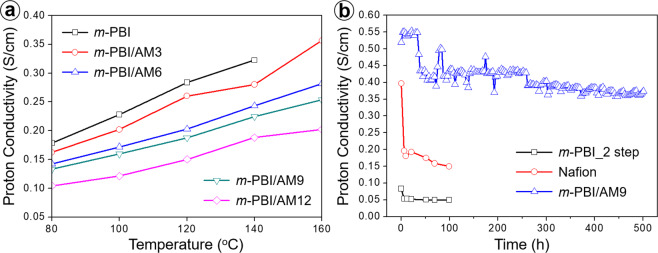
(**a**) Proton conductivity of acid-doped *m*-PBI composite membranes with different weight loadings of Al-MCM-41. (**b**) Comparison of the proton conductivity of Nafion, *m*-PBI, and *m*-PBI/AM9 membranes with respect to time at 150 °C.

Proton transfer through the membrane is a key parameter for the evaluation of the HT-PEMFC performance^45^. Figure 4(a) shows the proton conductivity of acid-doped *m*-PBI composite membranes measured at different temperatures in the range of 80–160 °C. The proton conductivity of the composite membranes increased with increasing operating temperature. The increase in temperature was beneficial for both proton transfer and structural reorganization. Thus, with the increase in temperature, both proton diffusion and molecular diffusion occurred, improving the proton conductivity. Additionally, the proton conductivity of the *m*-PBI membranes increased with the increasing Al-MCM-41 loading amount. The highest proton conductivity of 0.356 S cm^−1^ was achieved for *m*-PBI/AM9 whereas that of the pristine *m*-PBI was only 0.104 S cm^−1^. However, there were no data for *m*-PBI/AM12 at 160 °C owing to a measurement failure. This suggests that the durability of the specimen may have been reduced owing to the introduction of an excessive amount of filler into the PEM and the excess H_3_PO_4_ doping. Finally, the switched part of the sample may have been torn, which led to a data-plotting failure.

The fuel-cell performance of *m*-PBI/AM9 (the most stable and highest-performing composite membrane in this study) was compared to that of commercial Nafion and an *m*-PBI_2 step membrane prepared via a conventional two-step process (membrane synthesis and drying. followed by 3 d of H_3_PO_4_ doping), as shown in Fig. 4(b). The *m*-PBI_2 step and *m*-PBI/AM9 were measured at 150 °C and under non-humidified conditions, whereas the Nafion was measured at 80 °C and under humidified conditions because it had low thermal stability and needed water molecules as proton carriers. To compare the fuel-cell performances, the Nafion and *m*-PBI_2 step were measured for only 100 h. Their proton conductivity performance values were 0.15 and 0.05 S cm^−1^, respectively, under each operating condition. In contrast, the *m*-PBI/AM9 membrane was evaluated for 500 h to confirm its durability. The performance in the initial 100 h, mid-term 300 h, and end-of-life 500 h was evaluated. In the initial period, owing to the evaporation of water molecules, excessive H_3_PO_4_ leakage, and local dimensional changes in the electrolyte membrane, the proton conductivity decreased rapidly^46^. The mid-term performance declined by approximately 0.40 S cm^−1^, indicating the secondary stabilization of the electrolyte membrane. An additional change in performance due to phase separation occurred as excess free acid spontaneously leaked out of the electrolyte membrane. High performance of >0.35 S cm^−1^ was maintained even for long-term operation. In contrast to the previous proton-conductivity results, the proton conductivity of *m*-PBI/AM9 was approximately 6–7 times higher than that of the *m*-PBI_2 step. This was owing to the difference in the membrane fabrication processes. The *m*-PBI_2 step was prepared from the first production of dry film and doping with H_3_PO_4_ for 72 h to obtain an acid-doped membrane, whereas *m*-PBI/AM9 was fabricated via direct casting, which is a one-step sol-gel process. Therefore, in addition to the performance improvement owing to the unique process, a further improvement could have arisen from the introduction of the porous filler.

**Figure 5:**
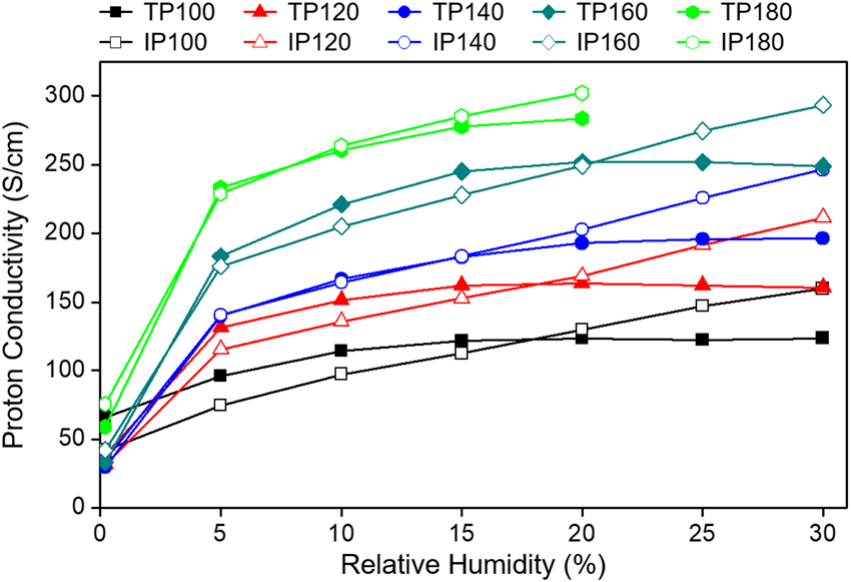
IP and TP proton conductivity of *m*-PBI/AM9 membranes under different temperature and relative humidity conditions.

The in-plane and through-plane conductivity values of the *m*-PBI/AM9 membrane were plotted, as shown in Fig. 5. “In-plane” (IP) refers to the proton conductivity at the surface of the electrolyte membranes, and “through-plane” (TP) refers to the proton conductivity when the proton passes through the electrolyte membrane. As indicated by the previous data (Fig. 4), only one type of value was gathered via the IP and TP measurements. Through this further analysis, IP and TP conductivity values were measured separately to compare the two methods with variations in temperature and humidity. First, both IP and TP conductivity improved as the temperature increased, which can be attributed to the increased proton transport activity at higher temperatures. In practice, PBI-based electrolyte membranes and their MEAs can exhibit normal properties only at temperatures of >150 °C, which tends to increase the activity of phosphoric acid as a proton carrier. By checking the conductivity when the humidity was changed from 0% to 30% for each temperature, it was possible to confirm the difference in the conductivity between IP and TP for a low humidity and high humidity. At a relatively low humidity (<15%), the TP conductivity was higher than the IP conductivity, whereas at a high humidity (≥20%), the IP conductivity tended to be higher than the TP conductivity. This phenomenon can be explained as follows. When the temperature was>100 °C and the relative humidity was <15%, the TP conductivity tended to be higher than the IP conductivity at the same temperature. Because of the insufficient water molecules that existed on the surface of the membrane during the high operating temperature, proton is efficiently transfer in the thickness direction. In contrast, at humidity values of ≥20%, the water molecules were relatively abundant on the surface of the electrolyte membrane, affecting the in-plane proton transport, as shown in the corresponding graph. According to the analysis results, when the *m*-PBI/AM9 membrane operates in a non-humidified condition at a high temperature above 150 °C, the TP conductivity is expected to be equal to or higher than the IP conductivity.

**Figure 6:**
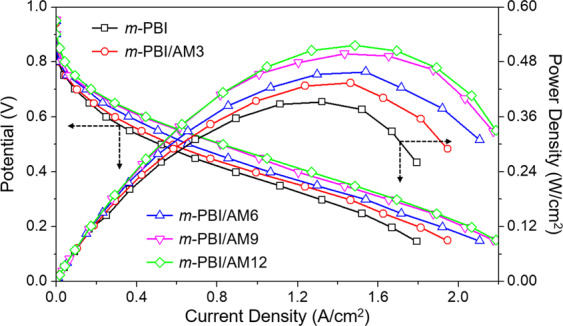
Combined fuel-cell polarization (I–V) and power density curves of *m*-PBI/Al-MCM-41 composite membranes at 150 °C with active area of 5 cm^2^.

Figure 6 and Table S3 show the MEA performance of *m*-PBI composite membranes with respect to the current density. According to an electrochemical analysis of the MEA, the polarization current–voltage (I–V) curve was plotted, and the power was calculated by multiplying the potential by the current. The *m*-PBI composite membranes exhibited similar open-circuit voltage (OCV) values for all the Al-MCM-41 loading amounts, whereas the current density and power density of all the membranes increased with the loading amount. The current density at 0.6 V and maximum power density of the *m*-PBI composite membranes increased from 0.251 and 0.446 A cm^−2^ to 0.393 and 0.516 W cm^−2^, respectively. All the parameters representative of the fuel-cell performance were significantly improved for the composite membranes because of the different preparation process, as confirmed by the proton-conductivity results. Additionally, the parameters were slightly improved for the composite membranes. The results indicated that the MEA performance of the *m*-PBI composite membranes was better than that of the pristine *m*-PBI. This enhancement in the MEA performance was a result of the introduction of the Al-MCM-41 channel, which had the capacity to hold H_3_PO_4_. Increased H_3_PO_4_ uptake led to an increased acid-doping phase in the membranes, a higher proton conductivity, and better MEA performance, and it improved properties directly related to the fuel-cell performance.

**Figure 7:**
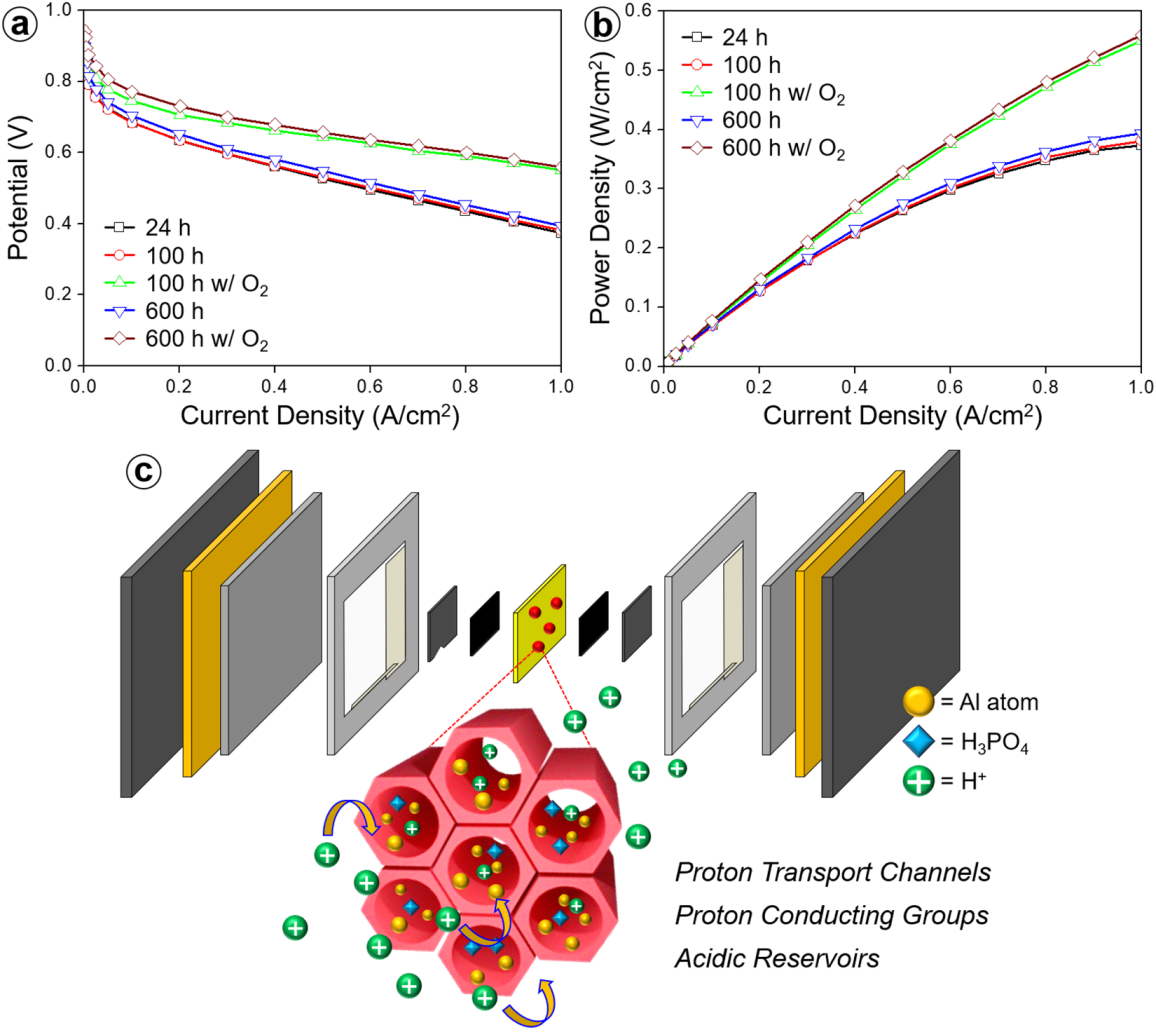
(**a**) Polarization (I–V) and (**b**) power density curves obtained before and after O_2_ enrichment for the *m*-PBI/AM9 membranes for different operating times. (**c**) Schematics of proton transfer path along the proton-conducting groups, proton-transport channels, and acidic reservoirs in *m*-PBI/Al-MCM-41 composite membrane system.

To investigate the long-term electrical properties of the electrolyte membranes, *m*-PBI/AM9 was tested for 600 h, and the polarization I–V curve was derived via electrochemical analysis at a specific time. According to the potential versus current density (Fig. 7(a)) and power density versus current density (Fig. 7(b)) results, the performance did not decrease but increased after 600 h. The data are similar to the proton-conductivity measurement results and indicate that the membrane featured stable performance and durability even under long-term operation at a high temperature. The I–V curves were obtained by supplying O_2_ instead of air to the cathode inlet at 100 and 600 h. Thus, pure O_2_ was used in the electrochemical reactions, enhancing the performance. According to the potential value of 0.6 V, after 100 and 600 h, the current density was 0.709 and 0.851 A cm^−2^, representing increases of 233 and 252% compared to those with air supply at the cathode side, respectively. This may have been due to the concentration of O_2_, which can cause an activity difference in the electrochemical reaction (99.9% pure O_2_ and air with an O_2_ volume concentration of 20%). The performance was improved for each series of composite membranes, and reliable durability and high performance were observed under long-term operating conditions. This result indicates that the proton conductivity can be improved by increasing the H_3_PO_4_ doping level, in agreement with previous reports. Thus, to improve the electrochemical performance of the electrolyte membrane, one-step process was adopted to obtain an H_3_PO_4_-doped membrane, and a “H_3_PO_4_-friendly” filler was incorporated into the *m*-PBI matrix (Fig. 7(c)).

In summary, an acid-doped PBI electrolyte composite membrane, comprising Al-MCM-41 channels as proton-carrier supports, was prepared via a one-step sol-gel transition using a direct casting method. To verify the improvement in the electrochemical properties, methodological approaches, such as TGA, proton conductivity, acid doping level, and I–V tests, were employed. The results confirmed that the performance of the composite membranes fabricated via direct casting followed by the sol-gel transition was superior to those of membranes prepared via conventional methods. Furthermore, the imidazole group with a H_3_PO_4_ affinity, as well as the well-ordered hexagonal honeycomb arrays of the highly porous structure, enhanced the electrochemical properties of the PEM. The proposed method is an effective and facile approach for the preparation of PEMs with high fuel-cell performance to satisfy the requirements of H_2_ energy applications.

## Methods

### Materials

3,3-diaminobenzidine (DAB, 99%), isophthalic acid (IPA, 99%), polyphosphoric acid (PPA, 115%), N,N-dimethylacetamide (DMAc, 99.5%), sodium silicate solution (~10.6% Na_2_O, ~26.5% SiO_2_), and aluminum chloride (AlCl_3_, 99.99%) were purchased from Sigma–Aldrich. Cetyltrimethylammonium bromide (CTABr) (>96%) was purchased from Fluka Co. Acetic acid (99.7%), ethyl alcohol (99.9%), and hydrochloric acid (HCl, 35% solution) were purchased from Duksan Chemical Co., Ltd. Pt/C (40 wt.% on carbon black) was purchased from Johnson Matthey. Deionized water was obtained from a Milli-Q Ultrapure water purification system.

### Preparation of Al-MCM-41

First, 3.25 g of CTABr was dissolved in 38 g of distilled water in a flask using a water bath at 40 °C. Then, 13.9 g of the sodium silicate solution was slowly added (dropwise) to the flask to induce the silicate reaction. Subsequently, the flask was placed in an oven at 100 °C, and the reaction proceeded for 24 h. The flask was then cooled, and the solution was titrated to a pH of 10 using a 50% acetic acid solution. The process of the reaction and titration was performed two more times, followed by washing with distilled water and filtration. The reaction product of the solid phase was placed in the oven and dried at 100 °C for 24 h. Then, the process of washing and filtering the sample using a solution containing 2.5 g of hydrochloric acid in 100 mL of ethanol was performed three times. The resultant product was dried and calcined at 550 °C in a furnace. AlCl_3_ was dissolved in ethanol for post-Al grafting, and 10 g of synthesized silica was added to the solution. The reaction proceeded for 24 h. The obtained solid powder was thoroughly washed with ethanol and filtered. Finally, the resultant product was sufficiently dried in the oven and calcined at 550 °C in the furnace for 4 h.

### Preparation of acid-doped m-PBI/Al-MCM-41 composite membranes

DAB and IPA with equal molar ratios were added to a 250-mL three-neck flask with a continuous N_2_ flow for flushing humidity and byproducts, followed by the addition of 200–400 g of PPA. The mixture was then agitated at 220 °C for 16 h, yielding a clear and viscous solution. The Al-MCM-41/PPA suspension was poured into the polymer solution and stirred for 3 h. The homogeneous solution was poured and cast onto a clean bare glass using a doctor blade with a gap height of 300–500 μm to achieve the sol-gel transition without a cooling process. Finally, the glass was placed in a chamber where the relative humidity was controlled at 55% and 25 °C to make the polymer solution absorb the water molecules for the sol-gel transition, resulting in a phosphoric acid (PA; H_3_PO_4_)-doped membrane.

### Phosphoric acid doping level calculation

The amount of acid doping was evaluated by weighting the membranes before and after the elimination of H_3_PO_4_. Acid-free undoped *m*-PBI composite membranes were prepared in DMAc instead of PPA process for quantitative evaluation of acid doping level. The prepared membranes were dried at 80 °C for 24 h under vacuum conditions, and their weights (*W*_dry_) were measured using a high-precision balance (OHAUS). The dried membranes were trimmed to 0.5 × 2 cm^2^ and then immersed in a H_3_PO_4_ solution. After 72 h, the membranes were removed from the container, and their surfaces were wiped. Then, the membranes were weighed (*W*_dop_), and the acid-doping level was calculated as follows:

1 $$Acid\,content=\frac{({W}_{dop}-{W}_{dry})}{{W}_{dry}}\times 100 \% ,$$

### Preparation of GDE and MEA

A gas-diffusion electrode (GDE) was prepared via the ink-spraying technique, as described in our previous work^37^. A catalyst solution was prepared by adding proper amounts of Pt/C (46.7 wt.% Pt, Tanaka Kikinzoku Kogyo K.K) and polytetrafluoroethylene (PFTE) binder (PFTE) (at a weight ratio of 4:1) to a water/IPA solution (1:1 w/w). The solution was sonicated for 30 min and sprayed onto SIGRACET GDL10 BC gas-diffusion layers. The catalyst loadings were evaluated by calculating the weight differences of the GDEs after the application of the catalyst inks. The prepared GDEs were annealed in a N_2_ atmosphere at 350 °C for 5 min. The Pt loadings of all the GDEs were controlled at 1.0 mg cm^-2^, with an active area of 5 cm^2^ for both cathode and anode electrodes. The membrane electrolyte assembly (MEA) was then prepared by pressing the GDEs against a H_3_PO_4_-doped membrane mounted on a gasket from both sides using a mechanical torque of 1 N at 120 °C for 10 min (Fig. S1).

### Measurements

Fourier transform infrared (FTIR) spectroscopy was implemented using FTIR − 4000 instruments in the range of 600–4000 cm^−1^. X-ray diffraction (XRD) patterns were obtained using a Miniflex X-ray diffractometer with Cu-Kα radiation (λ = 1.5405 Å) operating at 40 kV and 20 mA. Scanning electron microscopy (SEM) was implemented using a JSM-6701F field-emission scanning electronic microscope at 15 kV after Pt sputtering of each specimen surface. Transmission electron microscopy (TEM) was implemented using an energy-filtered JEM-2200FS. Thermogravimetric analysis (TGA) was conducted using a Q50 under a N_2_ flow at a heating rate of 20 °C min^−1^. The proton conductivity of the acid-doped membranes was measured via four-point electrochemical impedance spectroscopy over the frequency range of 10 Hz–400 kHz. The resistance was measured using an Autolab Impedence Analyzer with a conductivity cell, where the distance between the two electrodes was 1 cm. The conductivity was calculated as follows:

2 $$Conductivity(S/cm)=\frac{1}{\Omega }\times \frac{d}{A},$$

where Ω represents the resistance of the membrane, *d* represents the distance between the two electrodes, and *A* represents the electrode area. The flow rates at the anode (H_2_) and cathode (O_2_) were 150 ccm. The MEA performance of the membranes in a single cell was evaluated using a SMARTII cell station at 150 °C. For example, when the sample area was 1 cm^2^, the flow rates at the anode and cathode were 150 mL min^−1^.

## Supplementary information

Supplementary information.
