# Supplementary material for: Proton Transport in Aluminum-Substituted Mesoporous Silica Channel-Embedded High-Temperature Anhydrous Proton-Exchange Membrane Fuel Cells
Source: Sci Rep. 2020 Jun 25;10:10352. doi: 10.1038/s41598-020-66935-5 (PMC7316861; doi:10.1038/s41598-020-66935-5)
Supplement: Supplementary file 1 — Supplementary information. [file 41598_2020_66935_MOESM1_ESM.docx]

***Supplementary Information***

Proton Transport in Aluminum-Substituted Mesoporous Silica Channel-Embedded High-Temperature Anhydrous Proton-Exchange Membrane Fuel Cells

*Kwangwon Seo,‡ Ki-Ho Nam,‡ Haksoo Han**

*Department of Chemical and Biomolecular Engineering, Yonsei University, 50 Yonsei-ro, Seodaemun-gu, Seoul 120-749, Republic of Korea*


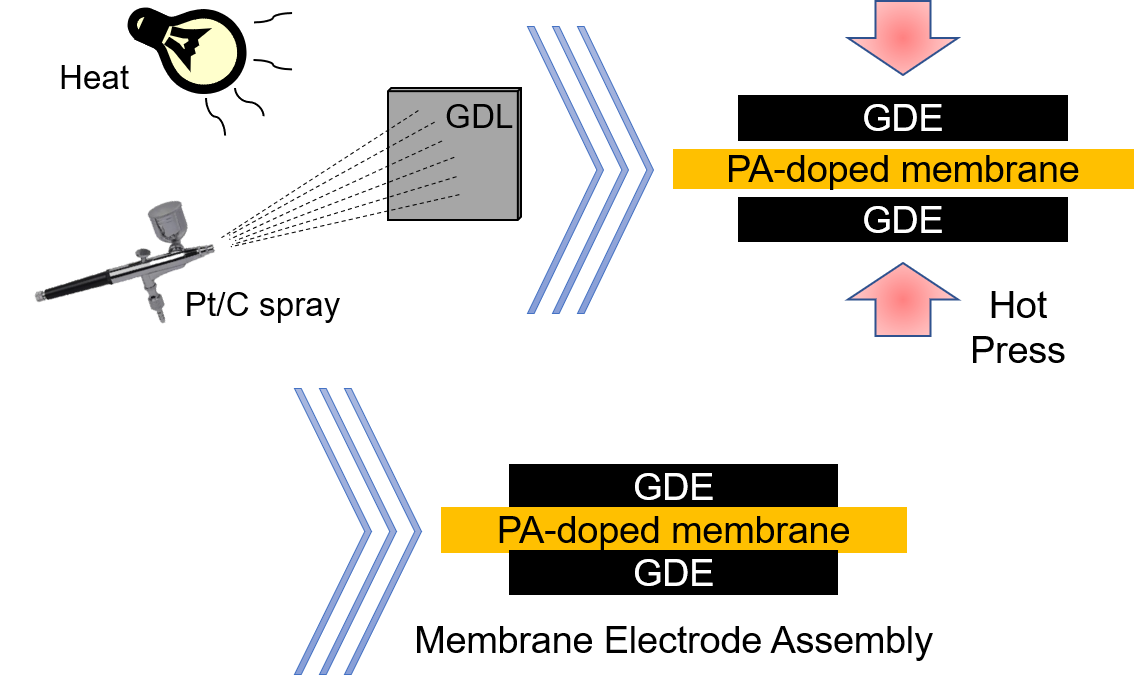


**Figure S1.** Fabrication process for the MEA.


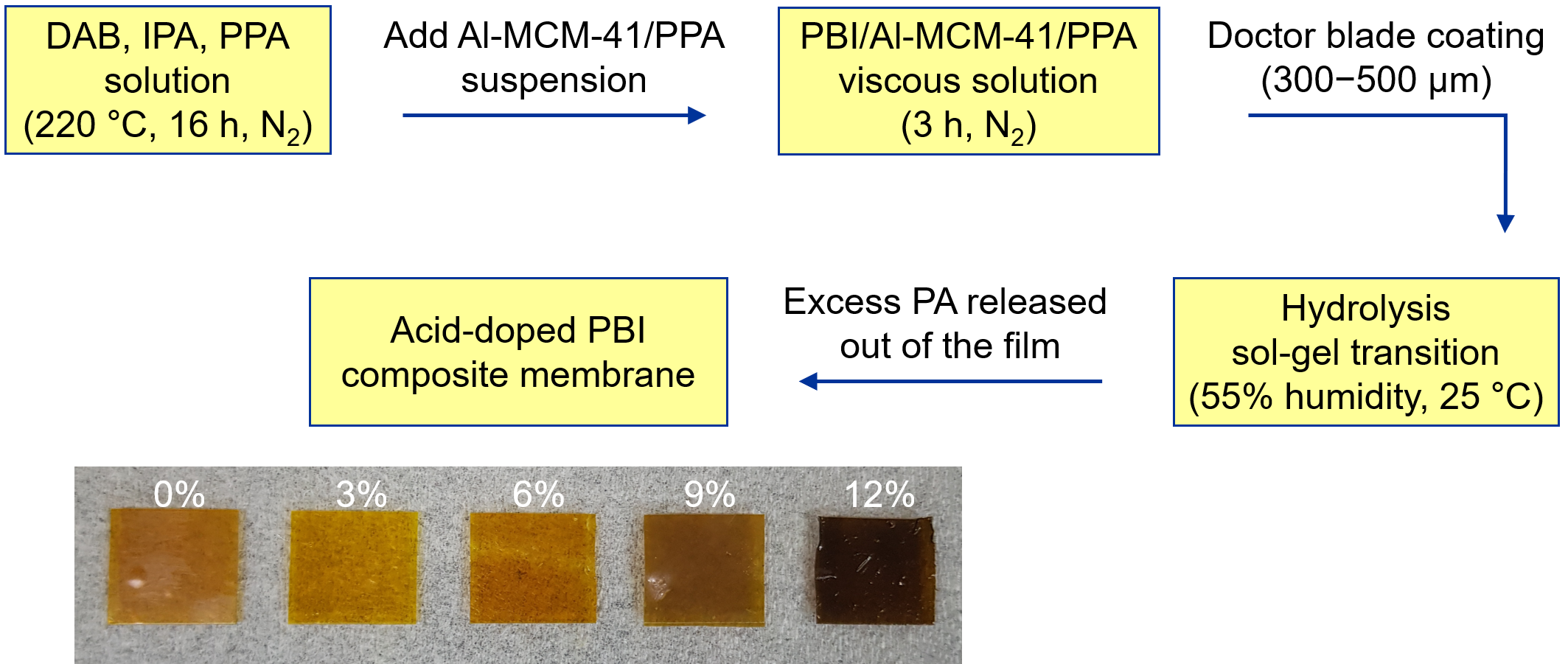


**Figure S2.** Acid-doped PBI composite membrane prepared by sol-gel transition.


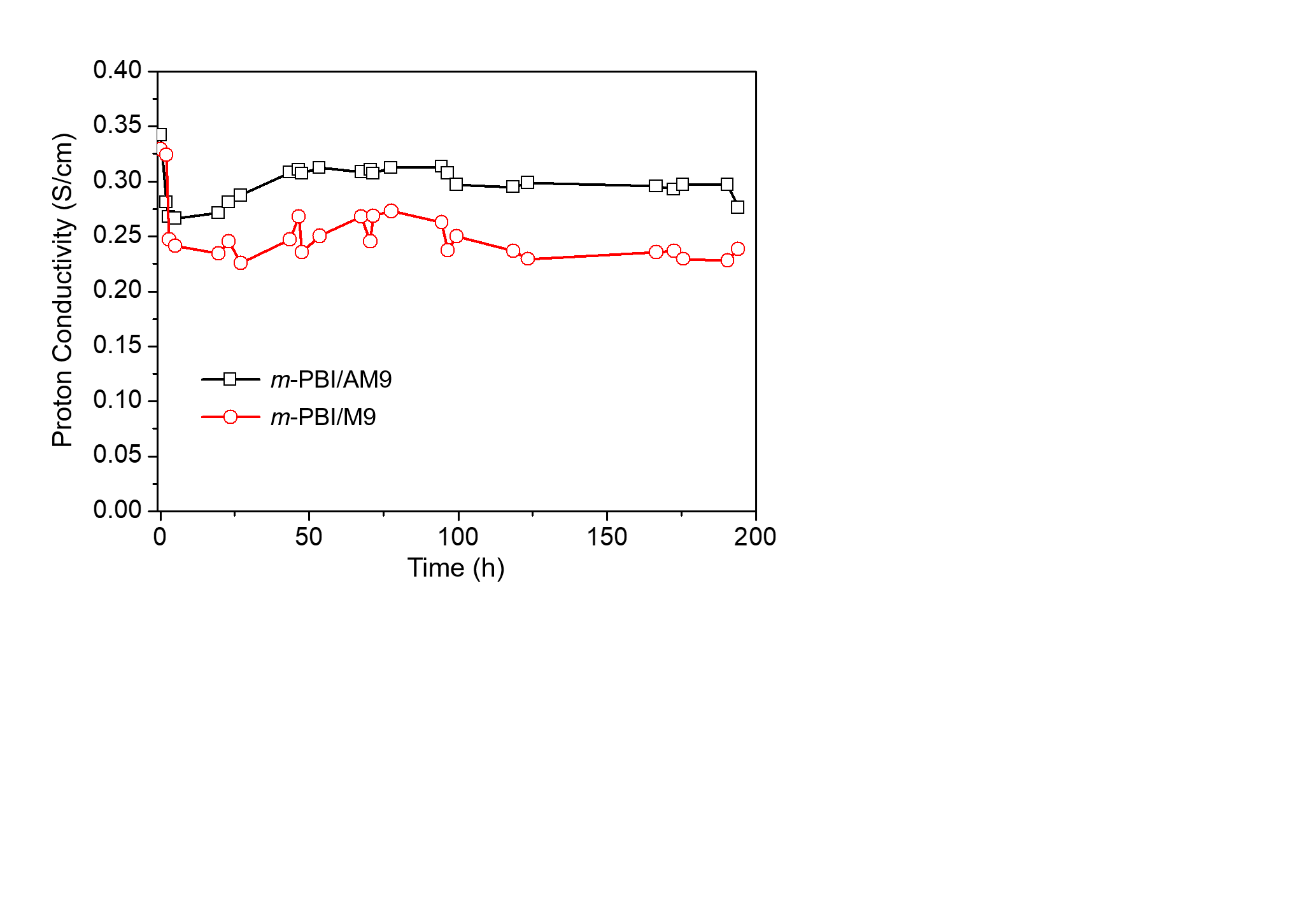


**Figure S3.** Comparison of the proton conductivity of *m*-PBI with 9 wt.% of Al-MCM-41 (*m*-PBI/AM9) and 9 wt.%MCM-41 (*m*-PBI/M9) membranes with respect to time at 150 °C.

Table S1. Thermal properties of acid-doped PBI composite membranes

| Membrane | *T_d_*_5%_ | *T_d_*_10%_ | Char residue at 600 °C |
| --- | --- | --- | --- |
| *m*-PBI | 420.3 | 576.2 | 85.2 |
| *m*-PBI/AM3 | 408.2 | 573.7 | 86.6 |
| *m*-PBI/AM6 | 382.3 | 558.7 | 87.8 |
| *m*-PBI/AM9 | 371.4 | 533.7 | 90.6 |
| *m*-PBI/AM12 | 349.7 | 523.7 | 91.6 |

Table S2. Acid-doping levels of *m*-PBI composite membranes

| Membrane | Sample size  (μm^2^) | Sample thickness  (μm) | Before doping  (g) | After doping  (g) | Amount of H_3_PO_4_  (g) | Amount of H_3_PO_4_ per specific volume  (g μm^−3^) | Acid doping level  (%) |
| --- | --- | --- | --- | --- | --- | --- | --- |
| *m*-PBI | 1.00 × 10^8^ | 20 | 2.41 × 10^−3^ | 6.11 × 10^−3^ | 3.70 × 10^−3^ | 1.85 × 10^−12^ | 153.5 |
| *m*-PBI/AM3 | 1.00 × 10^8^ | 20 | 2.44 × 10^−3^ | 9.23 × 10^−3^ | 6.79 × 10^−3^ | 3.40 × 10^−12^ | 278.3 |
| *m*-PBI/AM6 | 1.00 × 10^8^ | 20 | 2.42 × 10^−3^ | 1.19 × 10^−2^ | 9.48 × 10^−3^ | 4.74 × 10^−12^ | 391.7 |
| *m*-PBI/AM9 | 1.00 × 10^8^ | 20 | 2.39 × 10^−3^ | 1.29 × 10^−2^ | 1.05 × 10^−3^ | 5.26 × 10^−12^ | 439.7 |
| *m*-PBI/AM12 | 1.00 × 10^8^ | 22 | 2.56 × 10^−3^ | 1.49 × 10^−2^ | 1.23 × 10^−3^ | 5.61 × 10^−12^ | 482.0 |

Table S3. Electrochemical performance of *m*-PBI composite membranes

| Membrane | Pt catalyst loading  (mg cm^−2^) | OCV  (V) | Current density  at 0.6 V (A cm^−2^) | Maximum power density  (W cm^−2^) |
| --- | --- | --- | --- | --- |
| *m*-PBI | 1.5 | 0.921 | 0.120 | 0.196 |
| *m*-PBI/AM3 | 1.5 | 0.925 | 0.154 | 0.229 |
| *m*-PBI/AM6 | 1.5 | 0.936 | 0.210 | 0.268 |
| *m*-PBI/AM9 | 1.5 | 0.954 | 0.272 | 0.317 |
| *m*-PBI/AM12 | 1.5 | 0.978 | 0.320 | 0.370 |
